# Volumetric, Nanoscale Optical Imaging of Mouse and Human Kidney via Expansion Microscopy

**DOI:** 10.1038/s41598-018-28694-2

**Published:** 2018-07-10

**Authors:** Tyler J. Chozinski, Chenyi Mao, Aaron R. Halpern, Jeffrey W. Pippin, Stuart J. Shankland, Charles E. Alpers, Behzad Najafian, Joshua C. Vaughan

**Affiliations:** 1 Department of Chemistry, University of Washington, Seattle, Washington 98195 USA; 2 Department of Medicine, Division of Nephrology, University of Washington, Seattle, Washington 98195 USA; 3 Department of Pathology, University of Washington, Seattle, Washington 98195 USA; 4 Department of Physiology and Biophysics, University of Washington, Seattle, Washington 98195 USA

## Abstract

Although light microscopy is a powerful tool for the assessment of kidney physiology and pathology, it has traditionally been unable to resolve structures separated by less than the ~250 nm diffraction limit of visible light. Here, we report on the optimization, validation, and application of a recently developed super-resolution fluorescence microscopy method, called expansion microscopy (ExM), for volumetric interrogation of mouse and human kidney tissue with 70–75 nm lateral and ~250 nm axial spatial resolution. Using ExM with a standard confocal microscope, we resolve fine details of structures that have traditionally required visualization by electron microscopy, including podocyte foot processes, the glomerular basement membrane, and the cytoskeleton. This inexpensive and accessible approach to volumetric, nanoscale imaging enables visualization of fine structural details of kidney tissues that were previously difficult or impossible to measure by conventional methodologies.

## Introduction

The kidney glomerulus is a compact network of capillaries, supporting tissue, and resident cells. A critical structure of the glomerulus is the three-layered glomerular filtration barrier (GFB) that filters waste products from the blood space to the urinary space and is comprised of innermost fenestrated glomerular endothelial cells, glomerular basement membrane (GBM), and outermost interdigitated epithelial cells called podocytes. A major limitation in imaging the GFB is that many of the structures of the GFB are too small and too densely packed to be resolvable by the ~250 nm resolution of traditional diffraction-limited light microscopy. To date, the analysis of fine structural details of the GFB has primarily relied on electron microscopy (EM). Although extremely powerful, EM is typically limited to thin sections (<100 nm) and has a poor ability to report on distributions of specific protein molecules. Advanced EM methods such as serial block face scanning electron microscopy (SEM) or focused ion beam SEM can produce high-resolution volumetric image stacks that are on the order of 100 µm thick, although the instruments are not yet widely available and the data acquisition process is timeconsuming^1^. Correlative light and electron microscopy is technically demanding and requires use of sophisticated instruments and/or workflow, and typically lacks high-resolution volumetric information^2^. There is thus a strong need for new, accessible tools to interrogate kidney tissue with high spatial resolution, practical volumetric imaging capability, and molecular specificity.

A range of established super-resolution fluorescence microscopy methods are capable of analyzing 3D molecular distributions at length scales below 250 nm and have recently been applied to the study of kidney. Single molecule localization microscopy (SMLM), stimulated emission depletion (STED) microscopy, and structured illumination microscopy (SIM) have been used to study separate components of the GFB such as GBM composition^3^, slit diaphragm structure^4^, and podocyte effacement in diseased tissue^5^, respectively. Unfortunately, each of these methods currently suffers from certain limitations which still hinder widespread implementation. SMLM and STED have strict requirements for fluorophore properties, which create challenges for multicolor imaging. Additionally, SMLM, and to a lesser extent SIM (in its most common, commercial implementations) typically have poor resolution beyond a few micrometers from a coverglass substrate. Moreover, all of these methods require expensive, specialized instruments, and substantial technical and interpretive expertise.

**Figure 1:**
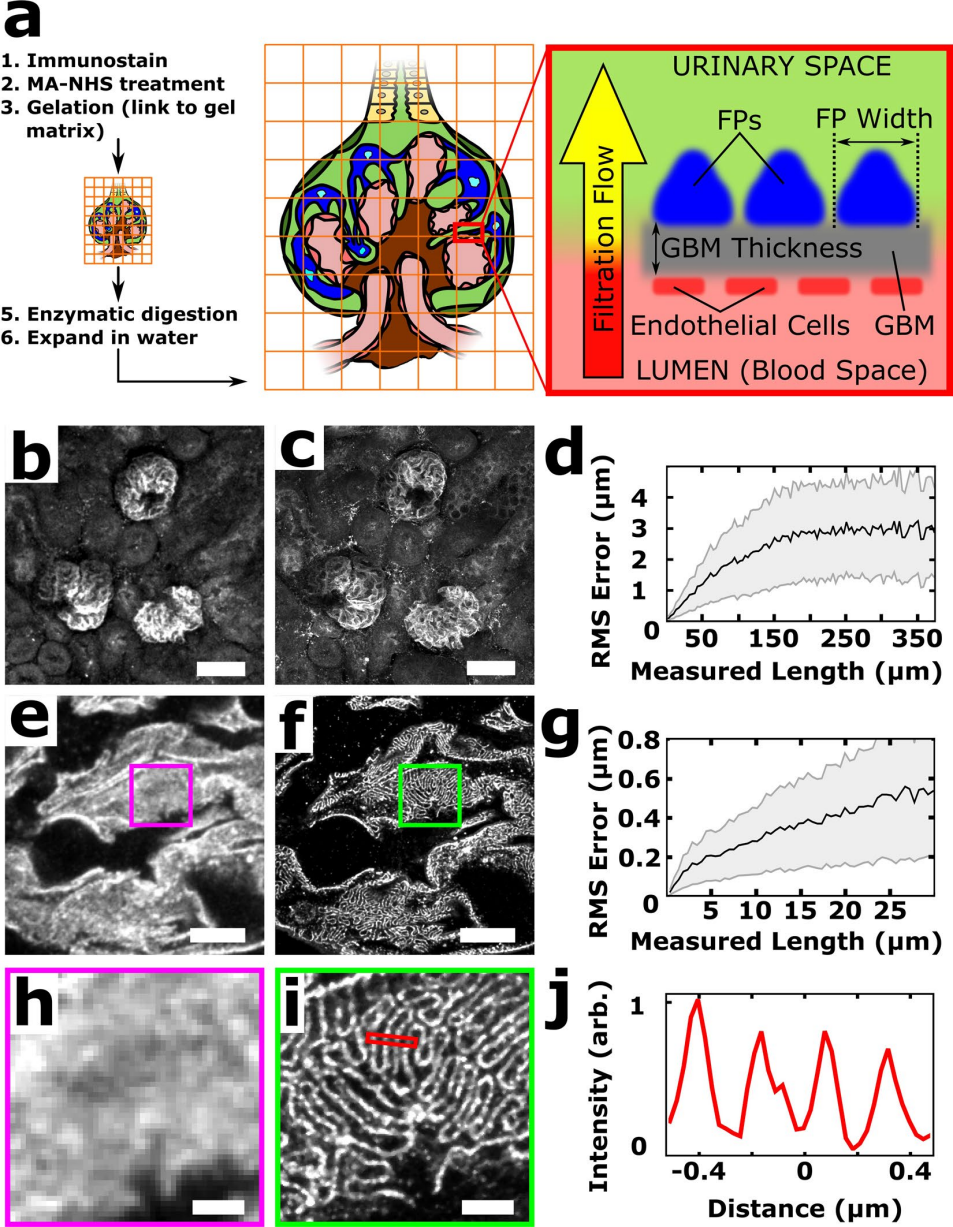
ExM schematic and validation in mouse kidney. (**a**) Fixed tissue is immunostained, treated with methacrylic acid *N*-hydroxy succinimidyl ester (MA-NHS) to covalently link antibody labels and proteins to the hydrogel during gelation, homogenized by enzymatic digestion, and then expanded ~4× along all dimensions by incubation with deionized water. (**b**–**c**) 100 μm thick mouse kidney section immunostained for podocin and imaged before (**b**) and after (**c**) expansion using a 10 × 0.4 numerical aperture (NA) air objective lens. (**d**) Quantification of expansion-induced distortions for panels b and c showing root mean square (RMS) error as a function of distance. (**e**–**f**) Mouse kidney section immunostained for podocin and imaged before (**e**) and after (**f**) expansion using a 63 × 1.2 NA water immersion objective lens. (**g**) Quantification of expansion-induced distortions for panels e and f. (**h**,**i**) Zoomed-in views of boxed regions in (**e**,**f**), showing that the expanded specimen successfully reveals interdigitated podocyte foot processes. (**j**) Cross-sectional profile of boxed region in (**i**). All distances and scale bars are in pre-expansion units. Scale bars, 50 µm (**b**,**c**), 5 µm (**e**,**f**), 1 µm (**h**,**i**).

Here, we report on the optimization, validation, and application of a recently developed super-resolution fluorescence microscopy method, called expansion microscopy (ExM), for volumetric nanoscale interrogation of mouse and human kidney tissue using a conventional fluorescence microscope. In ExM, fluorescent labels on fixed specimens are linked to a swellable polymer hydrogel that is grown within the specimen, after which the specimen is homogenized to facilitate uniform expansion and then swollen through incubation with deionized water (Fig. 1a)^6–9^. The physical magnification of the specimen in ExM allows features closer than the diffraction limit of light (~250 nm) to become resolvable in the expanded state. Additionally, the procedure renders samples optically clear with little scattering, facilitating deep volumetric imaging. With ~4× expansion per dimension, we achieve 70–75 nm lateral and ~250 nm axial spatial resolution at substantial depths, enabling nanoscale analysis of volumetric data sets as well as digital reorientation to ensure *en face* or orthogonal views. Additionally, because imaging is performed with a confocal microscope, multichannel data collection is straightforward.

## Results

Faithful expansion of a biological specimen using ExM requires the specimen to be homogenized after hydrogel embedding in order to avoid non-uniform expansion or tearing during the expansion process. Our initial results showed that homogenization procedures previously established for cultured cells and brain tissue using the broad-specificity protease proteinase K^6–8^ was unable to successfully homogenize fresh 100 μm mouse kidney sections embedded in hydrogel and led to gross distortions of the tissue (Supplementary Fig. 1). To test the hypothesis that the kidney’s collagen-rich extracellular matrix hindered homogenization, a collagenase digestion step was added and led to robust expansion with high fidelity (Fig. 1b-g, Supplementary Fig. 1). It should be noted that treatment with heat and detergent, which is used in the magnified analysis of the proteome (MAP) protocol under different fixation and hydrogel embedding conditions^9,10^, did not successfully homogenize the specimen (Supplementary Fig. 1). Interdigitated podocyte foot processes that were separated by <250 nm were clearly evident in the post-expansion images but were unobservable pre-expansion (Fig. 1e–f, h–j). A comparison of pre-expansion and post-expansion images of the same regions revealed that expansion-induced distortions were relatively minor, with 2–4% distortions for length scales up to ~150 μm and <2% for larger length scales (Fig. 1d,g; see also Supplementary Fig. 2). Based on the measured cross-sectional profiles of microtubules in expanded kidney tissue, we estimate our resolution to be ~70–75 nm (Supplementary Fig. 3). Note that all distances and scale bars for expanded specimens have been divided by their respective, measured expansion factors of ~4× and therefore refer to pre-expansion dimensions.

**Figure 2:**
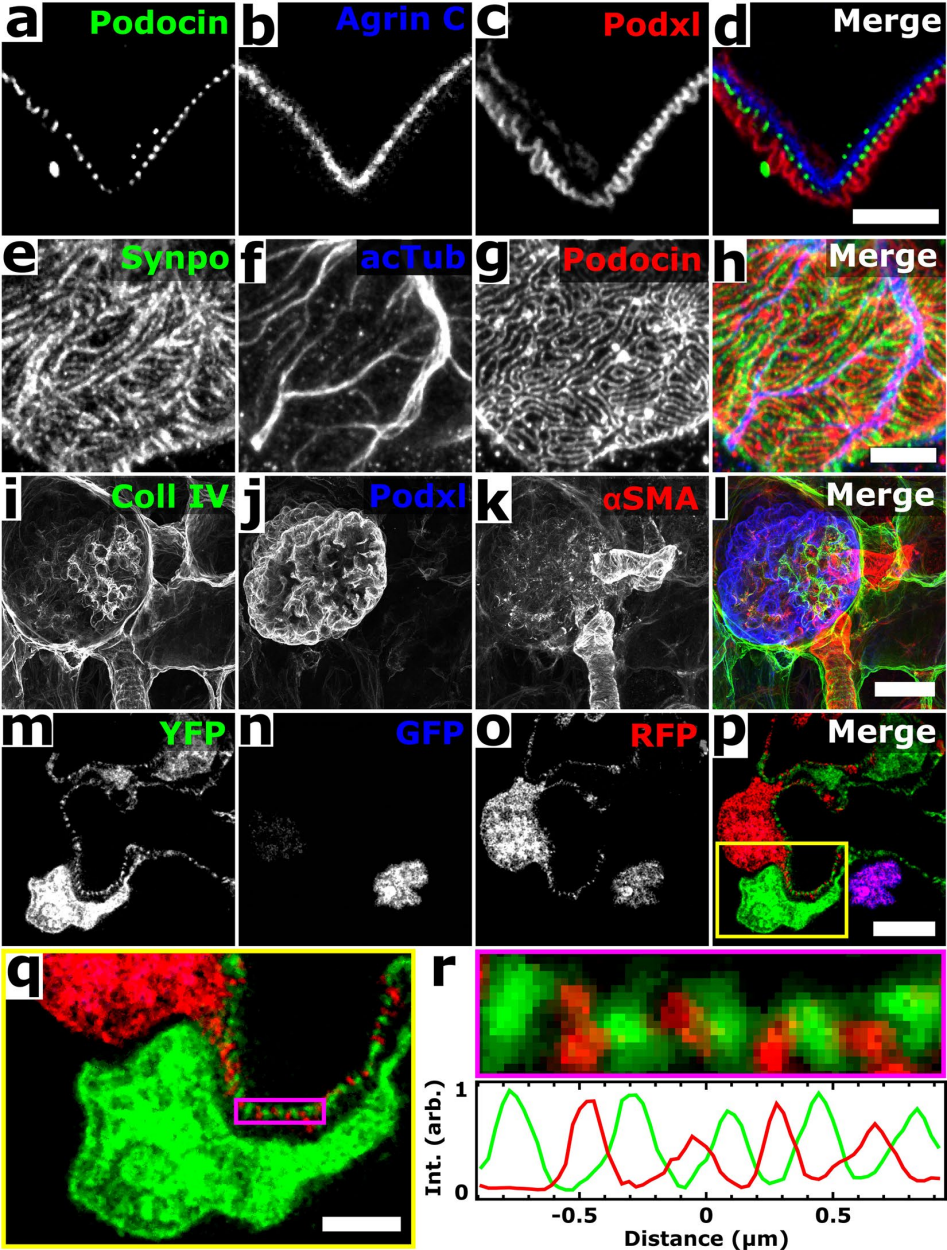
Confocal ExM images of mouse kidney labeled with antibodies or fluorescent proteins. (**a–c**) Single focal plane of glomerulus immunostained for podocin (**a**), agrin (**b**), podocalyxin (Podxl **c**), and merge (**d**) of (**a**–**c**). (**e–g**) Confocal maximum intensity projections of glomerulus immunostained for synaptopodin (Synpo, **e**), acetylated tubulin (acTub, **f**), podocin (**g**) highlighting secondary FPs, primary FPs, and slit diaphragms/FP boundaries, respectively. (**h**) Merge of (**e–h**). (**i**–**k**) Confocal maximum intensity projections of glomerulus immunostained for collagen IV (Coll IV, **i**), podocalyxin (Podxl, **j**), and α smooth muscle actin (αSMA, **k**) and highlighting Bowman’s capsule and the mesangium, podocytes, and arterioles and the mesangium, respectively. (**l**) Merge of (**i**–**k**). (**m**–**p**) Single focal plane of glomerulus showing native fluorescence from confetti mouse expressing YFP (**m**) and RFP (**o**) in separate podocyte cell bodies and FPs as well as GFP (**n**) in various podocyte nuclei. (**p**) Merge of (**m–o)**. (**q**) Zoomed-in view of region highlighted in (**p)**. (**r**) Further zoomed-in view (top) and cross-sectional profile (bottom) of boxed region highlighted in (**q)**. All distances and scale bars are in pre-expansion units. Scale bars, 2 µm (**a**–**h**,**q**), 25 µm (**i–l**), 5 µm (**m–p**).

We next assessed fine structural details that were observable by expanding mouse kidney sections that had been labeled with a range of antibodies or fluorescent proteins. A triple immunostain for podocin (podocyte foot process junctions), agrin (glomerular basement membrane), and podocalyxin (podocyte apical surfaces and, more weakly, endothelial cells) clearly resolved the separate layers of the GFB, and demarcated podocyte foot processes (Fig. 2a–d). A triple immunostain for synaptopodin (body of podocyte secondary foot processes), acetylated tubulin (podocyte primary foot processes, generally), and podocin enabled the identification of primary and secondary foot processes, and revealed the expected interior/surface relationship of stains for synaptopodin and podocin (Fig. 2e–h). A triple immunostain for full-length collagen IV (basement membrane, mesangium, and Bowman’s capsule), podocalyxin, and α smooth muscle actin (afferent and efferent arterioles and mesangium) showed larger-scale features of the glomerulus (Fig. i–l). Taking advantage of permanently lineage-tagged cells in reporter mice^11^, we found that our procedure is also compatible with genetically-encoded fluorescent proteins (without post-fixation immunolabeling) by recording images of expanded mouse kidney sections derived from a Podocin-confetti mouse (Fig. 2m–r). The expression of GFP, YFP, and RFP within different podocytes allowed them to be clearly identified as distinct cells over relatively large regions. Altogether, the expansion of kidney tissues was robust and versatile, with the ability to report on key glomerular structures and distributions of proteins using genetically encoded or exogenous fluorescent labels.

**Figure 3:**
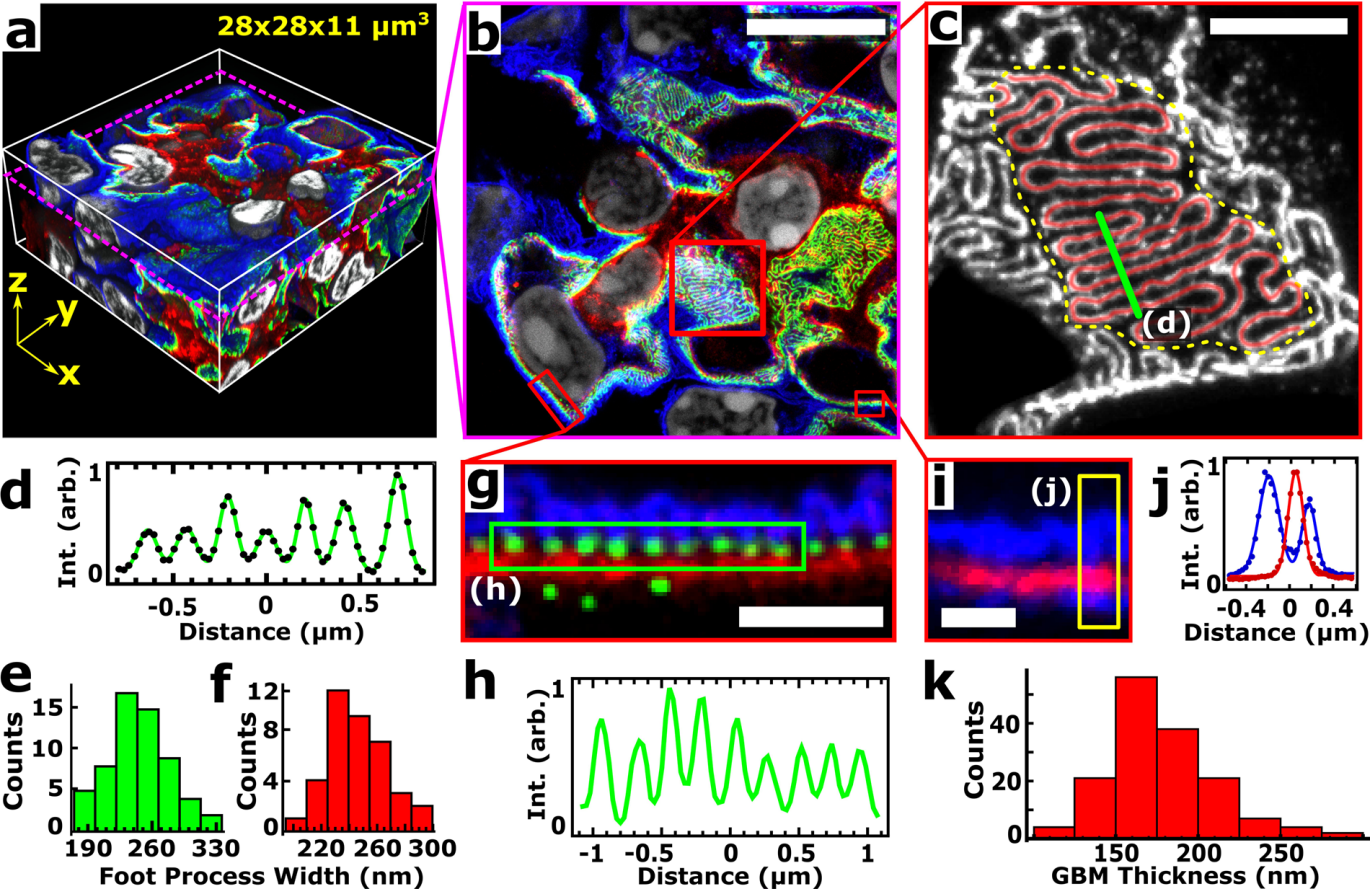
Quantification of GBM thickness and FP width. (**a**) Volume perspective rendering of expanded mouse kidney immunostained for podocin (green), agrin (red), podocalyxin (blue), and stained for DNA with Hoechst (white), highlighting podocyte foot process (FP) boundaries, glomerular basement membrane (GBM), endothelial cells, the apical side of FPs, and nuclei, respectively. See Supplementary Movie 1 for an animation of this volume. (**b**) A single confocal image plane from data shown in (**a)**. (**c**) Zoomed-in view of podocin channel from boxed region in (**b**) showing two methods for FP width (FPW) analysis. (**d**) Line profile (Int., signal intensity) of line in (**c**) (arb., arbitrary units) including a fit of the profile (black dots) with several Gaussians (green line) for determining positions of FPs. (**e**) Histogram of FPW determined by measuring peak-to-peak distances in (**d**) (data from 2 kidney samples, 7 glomeruli, and 61 profiles). (**f**) Histogram of average FPWs (data from 2 kidney samples, 7 glomeruli, and 39 profiles) determined by dividing the area within a flat region of the glomerulus (yellow dashed line) by the length of the podocin signal within that area (red lines in **c**). (**g**) Zoomed-in view of boxed region in (**b**) where the basement membrane and FPs are approximately perpendicular to the image plane (nuclear channel omitted for clarity). (**h**) Cross-sectional profile of the (green) podocin channel in the boxed region of (**g**). (**i**) Zoomed-in view of the podocalyxin (blue) and agrin (red) channels from the boxed region in (**b**). (**j**) Cross-sectional profile (points) of the boxed region in (**i**) together with Gaussian fits (lines). (**k**) Histogram of GBM thicknesses measured as the full width at half maximum of multiple agrin profiles (data from 3 kidney samples, 4 glomeruli, and 154 profiles). All distances and scale bars are in pre-expansion units. Scale bars, 8 µm (**b**), 2 µm (**c**), 1 µm (**g**), and 500 nm (**i**).

In order to further validate this approach, we measured quantitative features of the GFB such as foot process width and GBM thickness, which are metrics that are of clinical use and are typically obtained via transmission EM (TEM). Unlike TEM, however, which is typically measured in two dimensions on thin tissue sections, volumetric data sets are easily obtained with ExM (Fig. 3a, Supplementary Movies 1 and 2) and enable the viewing of images *en face* or orthogonally. This approach therefore avoids sectioning artifacts that commonly occur when imaging thin sections that can, for instance, lead to overestimation of foot process width or GBM thickness^12,13^. An immunostain for podocin was used to measure the average foot process width (FPW) in three different ways (Fig. 3c–h). First, an analysis of many cross-sectional profiles *en face* revealed an average peak-to-peak separation of 247 ± 29 nm (mean ± standard deviation (SD)), which was taken to be the average FPW (Fig. 3c–e). Second, the length of a cross-sectional profile drawn across an orthogonal view of podocin signal was divided by the number of foot processes (troughs) along it, giving an average FPW of ~250 nm (Fig. 3g,h). Third, a stereological approach originally developed for two-dimensional TEM^14,15^ was used to calculate the average FPW by dividing the area of a region of GFB by the total length of podocin signal within that area (red trace in Fig. 3c), again giving a similar value of 240 ± 46 nm (mean ± SD) (Fig. 3f). Previous studies utilizing different fixation and hydrogel-embedding methods^4,10^ from those used here have resolved the slit diaphragm and reported its width to be ~80 nm, close to our estimated spatial resolution of 70–75 nm. Next, the distribution of three GBM proteins were measured for regions of the GBM that were oriented perpendicular to the plane; agrin exhibited an average full width at half maximum (FWHM) of 178 ± 36 nm while vimentin and collagen IV stains in the same mouse revealed average GBM thicknesses of 192 ± 36 nm and 147 ± 26 nm (mean ± SD) (Supplementary Fig. 4), respectively, in agreement with previously measured GBM thicknesses measured by SMLM in ultracryomicrotome sections^3^. Nanoscale features could be resolved in these specimens at relatively large depths (up to ~120 µm in expanded specimens, or ~30 µm in pre-expansion units) and were limited here simply by the working distance of the objective lens available on our microscope Supplementary Figs 3, 5).

**Figure 4:**
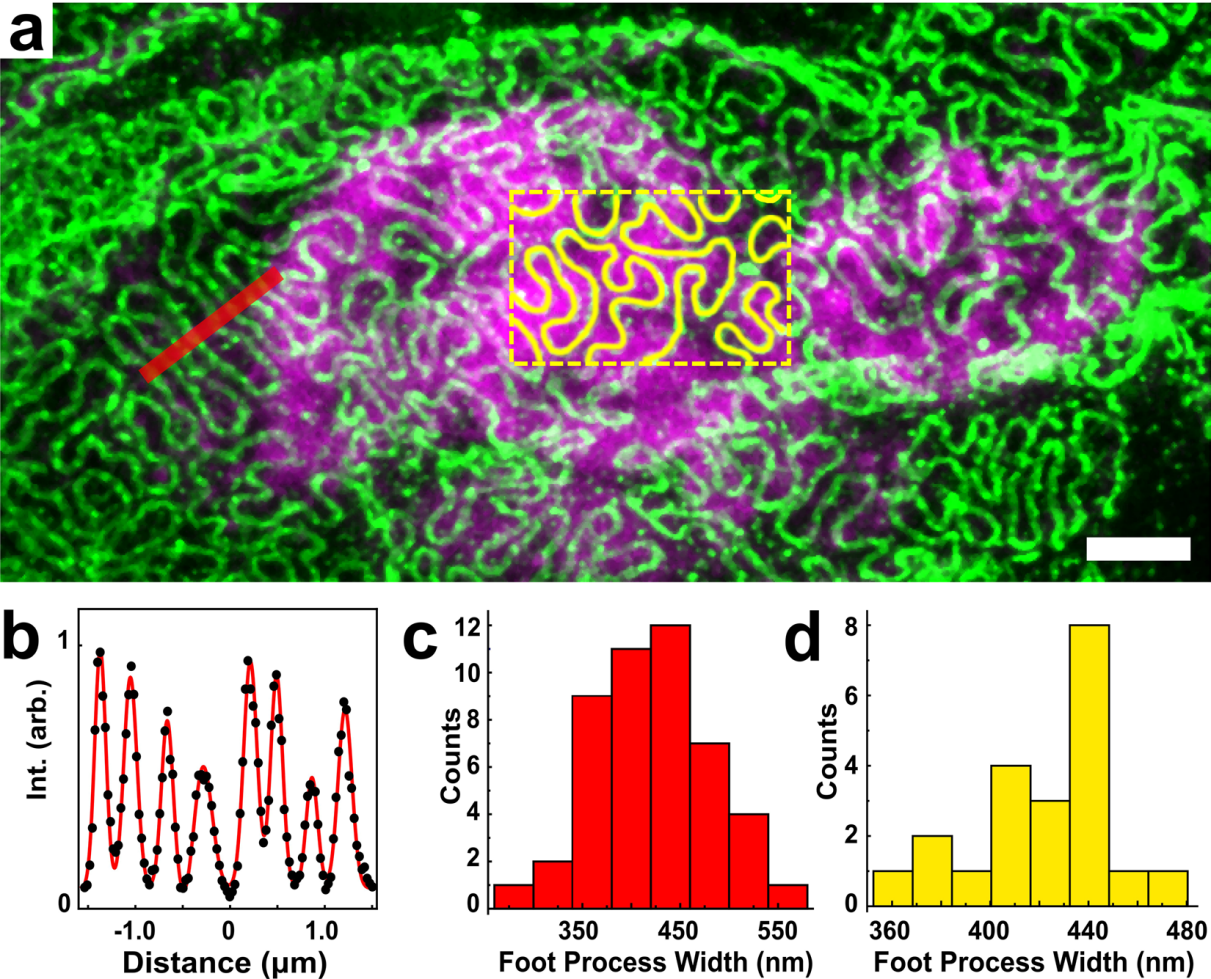
Confocal ExM images of expanded human kidney tissue. (**a**) Human kidney immunostained for podocin (green) with nuclei stained by Hoechst (magenta). (**b**) Cross-sectional profile along the red line in (**a**) and fitted with multiple Gaussian functions (black dots are data, and red curve is result of Gaussian fitting). (**c**) Histogram of foot process widths determined by a set of peak-to-peak distances in (**a**). (**d**) Histogram of foot process widths measuring by tracing podocin signal within a given area as in the yellow boxed region in (**a**), as described in the text. All distances and scale bars are in pre-expansion units. Scale bar, 2 μm (**a**).

We next sought to use the kidney ExM procedure described above on fresh human (nephrectomy) kidney tissue. Unfortunately, digestion with proteinase K and collagenase was insufficient for homogenization, and human kidney specimens expanded poorly (Supplementary Fig. 6). Based on our previous results showing that collagenase is necessary for mouse kidney digestion, we hypothesized that the poor homogenization was caused by the presence of one or more additional proteins in the extracellular matrix of human kidney which are more abundant than their counterparts in mouse kidney. We considered elastin as a candidate due to its abundance in human tissue^16–18^. Following the incorporation of an additional, initial digestion step with the enzyme elastase, in addition to the proteinase K and collagenase digestion steps, reliable expansion of fixed 100 μm human kidney sections was successfully achieved (Fig. 4a). Quantitative correlative analysis of pre- and post-expansion images of the same region of human kidney stained for podocin showed distortions of 1–3% over a range of length scales (Supplementary Fig. 7). Measurements showed foot process widths of ~418 ± 61 nm using a series of cross-sectional profiles (Fig. 4c) and ~426 ± 31 nm (mean ± SD) using areas divided by the length of the podocin signal (Fig. 4d), consistent with values reported in the literature^14,15^. In some instances, this methodology resolved two closely-spaced features within the podocin signal with a separation of 113 ± 13 nm (mean ± SD) (Supplementary Fig. 8) that likely represent expression of podocin within adjacent foot processes.

## Discussion

Our results show the optimization and validation of new ExM protocols which enables volumetric, nanoscale imaging of mouse and human kidney tissue using a standard confocal microscope. Importantly, the procedures utilize new homogenization strategies to enzymatically digest the extracellular matrix of mouse and human kidney tissue with minimal distortion upon expansion (1–4%). These or other tissue-optimized digestion procedures may also be useful for ExM with other tissues whose substantial extracellular matrix or other components may also resist enzymatic digestion. While electron microscopy has vastly superior spatial resolution compared to ExM, the 70–75 nm resolution demonstrated here is sufficient to resolve key nanoscale structures important for understanding kidney function in the laboratory or clinic while also providing the ability to measure 3D distributions of multiple, specific molecules. These 3D data sets allow users to identify areas of interest with a desired orientation (i.e. *en face*, orthogonal, etc.) which helps to avoid sectioning artifacts and thereby simplifies analysis and interpretation. Additionally, the simplicity and reduced cost of ExM compared to EM or other optical super-resolution techniques, makes the technique accessible to a wide range of potential practitioners.

Three recent studies have also applied expansion methodology to the kidney. Zhao *et al*. applied ExM to the study of ~5 μm thick formalin-fixed paraffin-embedded (FFPE) tissue sections and was limited to two-dimensional imaging^19^. The FFPE kidney sections expanded with low distortion in the Zhao *et al*. study^19^ using only proteinase K digestion, perhaps due to some combination of a low amount of connective tissue in the very thin specimens and/or structural changes to the specimen due to FFPE sample processing (e.g., dehydration or treatment with organic solvents, etc.)^20^. Ku *et al*. demonstrated expansion of multiple, perfusion-fixed whole mouse organs including kidney but only imaged and quantitatively validated the procedure with brain and cultured cells^9^. During the final stages of this work, a similar procedure to that of Ku *et al*., but different from ours, was reported by Unnersjö-Jess *et al*.^10^ for expansion of rat and mouse kidney, and the authors demonstrated the nanoscale, volumetric imaging of rat and mouse glomeruli using specimen expansion with confocal microscopy.

We note a number of key differences between the Unnersjö-Jess *et al*. study and ours, which we believe are complementary studies. First, we performed careful imaging of kidney tissues both before and after expansion in order to quantify the amount of distortion introduced by expansion, and we used this to show quantitatively that our procedures induce minimal distortion over a range of length scales for both mouse and human kidney tissues. In contrast, Unnersjö-Jess *et al*. compared two average feature sizes from different samples before and after expansion, although their results were strong on a qualitative level and no obvious distortions were apparent. Second, while our immunolabeling was performed prior to gelation, the procedure of Unnersjö-Jess *et al*. used immunolabeling after expansion. Thus, pre-expansion and post-expansion labeling may have different abilities to detect epitopes, and there is precedence for some epitopes being masked due to hydrogel polymerization and others that are revealed upon denaturation^9^. Additionally, post-expansion staining may cause less antibody-induced broadening since pre-expansion labeling leads to expansion of the antibody labels while post-expansion labeling does not^8^. Third, our procedures enabled us to image intrinsic signal from fluorescent proteins in kidney in reporter mice, including ones which are not antigenically distinct (i.e., GFP, YFP, etc.), while the Unnersjö-Jess *et al*. protocol did not. Fourth, using five-fold expansion together with deconvolution confocal or STED microscopy, Unnersjö-Jess *et al*. were able to achieve higher spatial resolution than we achieved here with four-fold expansion and standard confocal microscopy, although these techniques could in principle also be implemented with our procedures to similarly improve the spatial resolution. Lastly, we have provided extensive documentation of our procedures for ExM of mouse and human kidney tissue in a detailed experimental protocol (Supplementary Protocol 1) to help accelerate the dissemination of these methods for a range of applications from the research laboratory to the clinic.

## Materials and Methods

### Reagents and Reagent Preparation

Unconjugated secondary antibodies were obtained from Jackson Immunoresearch (West Grove, PA, USA) and included donkey anti-mouse (715-005-151), donkey anti-rabbit (711-005-152), donkey anti-goat (705-005-147), donkey anti-rat (712-545-150), and donkey anti-guinea pig (706-005-148). Primary antibodies were purchased as follows: Rabbit anti-podocin (P0372, Sigma-Aldrich, St. Louis, MO, USA), goat anti-podocalyxin (AF1556, R&D Systems Inc., Minneapolis, MN, USA), mouse anti-agrin (6D2, DSHB, Iowa City, IA, USA), rabbit anti-collagen IV (ab6586, Abcam, Cambridge, MA, USA), mouse anti-acetylated tubulin (T7451, Sigma-Aldrich), mouse anti-alpha smooth muscle actin (904601, BioLegend, San Diego, CA, USA), guinea pig anti-synaptopodin (internal N-terminus) (GP94-IN, Progen Biotechnik, Heidelberg, Germany), mouse anti-vimentin (18-0052, Thermo Fisher Scientific/Invitrogen, Waltham, MA, USA), and rat anti-alpha tubulin (Thermo Fisher Scientific). NHS-functionalized dyes were purchased from Sigma-Aldrich (ATTO 488 and ATTO 647 N) and Thermo Fisher Scientific (Alexa Fluor 488 and Alexa Fluor 568). NHS-functionalized dyes were received in 1 mg aliquots and were dissolved at a concentration of 100 mg/mL in anhydrous DMSO, further diluted into subaliquots of 1–10 mg/mL, and were stored at −20 °C. Hoechst 33342 was purchased from Life Technologies (Carlsbad, CA, USA) and was used according to manufacturer’s instructions. NAP-5 size-exclusion chromatography columns were purchased from GE Healthcare (Little Chalfont, Buckinghamshire, United Kingdom). Methacrylic acid N-hydroxy succinimidyl ester (MA-NHS) was purchased from Sigma-Aldrich and was dissolved in anhydrous DMSO in 1 M aliquots which were stored at −20 °C. Paraformaldehyde (32%) was obtained from Electron Microscopy Sciences (Hatfield, PA, USA). Tetramethylethylenediamine (TEMED, 17919) and ammonium persulfate (APS, 17874) were obtained from Thermo Fisher Scientific. 4-hydroxy-2,2,6,6-tetramethylpiperadin-1-oxyl (TEMPO, 97%, 176141) and sodium acrylate (97%, 408220) were purchased from Sigma Aldrich. 40% acrylamide (1610140) and 2% bis-acrylamide (1410142) were obtained from Bio-Rad Laboratories (Herculues, CA, USA). Proteinase K was purchased from Thermo Fisher Scientific (EO0491), collagenase (F type blend, C7926) from Sigma-Aldrich, and elastase (16-19-051200-porcine) from Athens Research & Technology (Athens, GA, USA). 10x phosphate buffered saline (70011044) and 10x Tris-acetate-EDTA (TAE, 15558042) buffers were purchased from Thermo Scientific. Hank’s Balanced Salt Solution (HBSS, 1x) buffer was purchased from Corning (Manassas, VA, USA). Bovine serum albumin (BSA) was obtained from Santa Cruz Biotechnology (Santa Cruz, CA, USA).

### Preparation of fluorophore-labeled secondary antibodies

Fluorescent dyes were coupled to secondary antibodies in-house by first mixing ~40 µL of unconjugated antibody (~1.3 mg/mL), 5 µL of 1 M sodium bicarbonate (pH ~8.3), and 1–2 µL of NHS dye (stocks made in DMSO at 1–10 mg/mL depending on the dye). The reaction was allowed to proceed at room temperature for 30 min. For purification, a NAP-5 size exclusion column was equilibrated by flowing through 10–20 mL PBS. The entire reaction mixture was then loaded onto the column followed by 650 µL of PBS which was discarded after elution. Another 300 µL was then added and the eluent was collected. Characterization of the dye-to-IgG ratio was performed with UV/Vis absorption spectroscopy and the results can be found in Supplementary Table 1.

### Fluorescence microscopes

Conventional epifluorescence microscopy was performed with an inverted Nikon Ti-S microscope fitted with a 4 × 0.2 NA air objective lens (Nikon, Melvill, NY, USA), a 10 × 0.25 NA air objective lens (Nikon), or a 20 × 0.45 NA air objective lens (Nikon). Illumination was achieved using a four-channel light emitting diode (LED4D120, Thorlabs, Newton, NJ, USA) with a multiband filter set LF405/488/532/635-A-000, Semrock, Rochester, NY, USA) and images were captured using a Zyla 5.5 sCMOS camera (Andor, Windsor, CT, USA). Confocal microscopy was performed on a Leica SP5 inverted confocal point scanning microscope at the UW Biology Imaging Core using a 10 × 0.4 NA air objective lens (Leica, Nussloch, Germany), 20 × 0.7 NA air objective (Leica), and a 63 × 1.2 NA water immersion objective (Leica).

### Mouse kidney dissection and preparation

All experimental protocols and methods in this work involving animals were approved by and conducted in accordance with all guidelines and regulations set forth by the Institutional Animal Care and Use Committee at the University of Washington. At approximately 2 months old, female mice (strain C57BL/6) were sacrificed by suffocation with CO_2_ followed by cervical dislocation. Kidneys were immediately removed and the renal capsule was stripped off. The kidneys were then halved longitudinally and immersed in 4% PFA in PBS for 1 hour at room temperature. They were then washed with PBS and sliced to 100 µm thickness using a vibratome. The slices were stored in PBS at 4 °C until staining.

### Confetti-mouse kidney dissection and preparation

All experimental protocols and methods in this work involving animals were approved by and conducted in accordance with all guidelines and regulations set forth by the Institutional Animal Care and Use Committee at the University of Washington. NPHS2Cre/R26R-ConfettiTG/WT (podocin/cre recombinase/confetti) mice were used to provide permanent and stochastic expression of one of four fluorescent colored reporters in podocytes. These colors were cytoplasmic targeted red fluorescent protein (cRFP), membrane targeted cyan fluorescent protein (mCFP), nuclear targeted green fluorescent protein (nGFP) or cytoplasmic targeted yellow fluorescent protein (cYFP). Mice were sacrificed with an overdose of Ketamine/Xylazine, cardiac perfused with 10–15 mL of ice cold phosphate buffered saline (PBS) at 25 inches of gravity pressure (47 mmHg or 63 mbar) through a 21 G butterfly infusion set (UW IACUC 2968–04). Kidneys were placed into 4% paraformaldehyde in PBS (PFA, Affymetrix, Santa Clara, CA) for 45 minutes, washed briefly in PBS, placed in 30% sucrose in PBS (Sigma-Aldrich, St Louis, MO) overnight, blotted dry, embedded in Tissue-Tek® O.C.T. Compound (VWR, Radnor, PA), and frozen in a 100% ethanol/dry ice bath. Kidney slices (50 µm thickness) were obtained using a Leica CM1850 cryostat, placed into 24-well culture dish and stored at −80 °C until staining. Tissue slices were fixed for 1 h in 3% PFA in PBS at room temperature for 1 h and then washed with PBS prior to immunostaining.

### Human kidney tissue preparation

De-identified non-tumoral human kidney tissue samples from individuals undergoing nephrectomy for a kidney tumor and no history of other kidney disease were obtained from NW Biotrust under a protocol approved by the University of Washington Institutional Review Board with informed patient consent. All experimental protocols and methods involving human tissue were performed in accordance with the guidelines and regulations set forth by the University of Washington Institutional Review Board. The samples were cut to be ~1 mm^3^ and immersed in 4% PFA in PBS for 1 h at room temperature. The tissue was then washed with PBS and sliced to 100 µm thickness using a vibratome. The slices were stored in PBS at 4 °C until staining.

### Tissue immunostaining and pre-expansion MA-NHS treatment

Both human and mouse kidneys were stained using the following protocol. 100-µm-thick kidney slices were first incubated in blocking/permeabilization buffer (3% BSA and 0.1% Triton X-100 in PBS) for at 6–12 h at 4 °C. The slices were then incubated with primary antibody diluted in blocking/permeabilization buffer (see Supplementary Table 1 for dilutions) for 18–24 h at 4 °C and were then washed with blocking/permeabilization buffer three times at room temperature (15 min each). The tissues were then incubated in fluorescently labeled secondary antibodies diluted in blocking/permeabilization buffer for 18–24 h at 4 °C. The slices were then washed three times with PBS at room temperature (15 min each). Nuclear staining was performed with Hoechst 33342 according to manufacturer instructions. Tissues were then incubated in 1 mM MA-NHS in PBS (diluted freshly from a 1 M stock in DMSO) for 1 h at room temperature followed by three washes with PBS.

### Tissue Gelation

After immunostaining and treatment with MA-NHS, the tissue slices were incubated in monomer solution (1× PBS, 2 M NaCl, 2.5% (wt/wt) acrylamide, 0.15% (wt/wt) N,N′-methylenebisacrylamide, 8.625% (wt/wt) sodium acrylate) for at least 1 h at 4 °C. The tissues were then placed on a clean #1.5 coverglass and the excess liquid was removed from the sample. A concentrated stock of TEMPO at 1% (wt/wt) in water was diluted in monomer solution to a final concentration of 0.01% (wt/wt). TEMPO acts as an inhibitor to the gelation reaction to allow complete diffusion of the monomers through the tissue. Concentrated stocks of APS and TEMED at 10% (wt/wt) in water were also diluted alongside the TEMPO in monomer solution to final concentrations of 0.2% (wt/wt) with APS added last. The gelation solution was gently applied to the tissue so that the sample did not leave the surface of the glass. Two stacked small pieces of #1.5 coverglass were placed on either side of the sample and another whole coverglass was placed on top to create a tissue gelation chamber. Tissues were incubated in a humidified environment at 37 °C for 1.5–2.5 h. The chambers were disassembled leaving the gelled sample on one piece of coverglass. The excess gel and glass around the sample was cut and removed for digestion.

### Mouse kidney digestion, expansion, and nuclear restaining

Gelled mouse kidney samples were transferred to at least 1 mL of proteinase digestion buffer (1× TAE buffer, 0.5% Triton X-100, 0.8 M guanidine HCL) containing 8 units mL^−1^ proteinase K and were digested at 37 °C overnight. The gels were then washed in PBS and transferred to collagenase buffer (1× HBSS + 0.7 mM CaCl_2_) with 5 mg mL^−1^ collagenase (at least 1 mL total volume). Next, the samples were digested overnight at 37 °C. After digestion, the samples were placed in DI water to expand (typically 30 min with 1–2 water exchanges). Nuclear staining with Hoechst 33342 was performed again (using water as a solvent) at this stage as well. It should be noted that larger samples may require larger volumes of digestion solutions for complete homogenization.

### Human kidney digestion, expansion, and nuclear restaining

Gelled human kidney samples were transferred to at least 1 mL of 200 mM Tris buffer (pH 8) containing 1 mg mL^−1^ elastase and were allowed to digest at 37 °C overnight. The samples were washed with PBS and transferred to proteinase digestion buffer containing 8 units mL^−1^ proteinase K. The samples were digested at ~65 °C overnight. The digestion buffer and proteinase were removed and refreshed the next day and the samples were allowed to again digest overnight at 65 °C. After washing with PBS, the samples were digested overnight at 37 °C in collagenase buffer with 5 mg mL^−1^ collagenase. The samples were then transferred to DI water for expansion. Nuclear staining with Hoechst 33342 was performed again (using water as a solvent) at this stage as well.

### Expanded sample handling

The gels were immobilized on glass as follows. Rectangular #1.5 coverglasses were plasma cleaned and coated with poly lysine by spreading an aqueous solution of poly lysine over their surface and allowing them to air dry. Gels were cut to fit on the rectangular coverglass and the excess water was removed by wicking it away from the edges with tissue paper. The gels were then gently placed onto the poly lysine glass by sliding the gel off an untreated piece of glass using a low contact angle with respect to the treated glass to avoid sample bending. The immobilized gels were then immediately imaged.

### Image Visualization

Fiji and Imaris (Bitplane) were used for visualization of all images acquired in this study.

### Correlative image analysis

Quantitative distortion analysis was performed as described previously^7^. Briefly, pre- and post-expansion images of the same region were first aligned using a rigid transformation (similarity, linear) to roughly align the images and determine the expansion factor. Next, a non-rigid (B-spline, non-linear) transformation was applied to the post-expansion image so that it matched the pre-expansion image (see also Fig. S2).

### Foot Process Width Measurement by Tracing Method

Areas of expanded, podocin-stained tissue samples were first isolated based on their flatness in the image plane. Areas that remained within a thickness of 0.75 µm (distance in pre-expansion units) were chosen for further analysis while others were rejected to avoid projection artifacts. The podocin signal in the maximum intensity projections of the selected areas were then traced in ImageJ and the length of the traces was recorded. Dividing the area of analysis by the length of trace it contained gave the average foot process width.

### Foot Process Width Measurement from Cross-Sectional Profiles

Using the above method to ensure the area of interest was oriented flat with respect to the image plane, we obtained line profiles in the podocin channel of expanded kidney specimens. Line profiles that were perpendicular to the foot processes were chosen for analysis. The adjacent peak-to-peak distance was measured after fitting the profiles with Gaussian functions to obtain foot process width values.

### Glomerular Basement Membrane Thickness Measurement

Expanded specimens stained for podocalyxin and a GBM marker (agrin, collagen IV, or vimentin) were used for this analysis. Orthogonal image sections were chosen using the podocalyxin channel. Podocalyxin stains both the apical side of foot processes as well as endothelial cells, and we used the lack of lateral movement of these features when scrolling through confocal image stacks to visually confirm the area of interest was perpendicular to the image plane in order to avoid projection artifacts. A line profile perpendicular to the GBM and spanning its entire thickness was drawn and the cross-sectional profile obtained from the GBM marker channel (either agrin, collagen IV, or vimentin) was fitted with a Gaussian function whose full width at half maximum was taken to be the GBM thickness.

### Data Availability

The datasets that support the findings of this paper are available from the corresponding author upon request.

## Electronic supplementary material

Supplementary Movie 1

Supplementary Movie 2

Supplementary Information and Protocol
