# Supplementary material for: Volumetric, Nanoscale Optical Imaging of Mouse and Human Kidney via Expansion Microscopy
Source: Sci Rep. 2018 Jul 10;8:10396. doi: 10.1038/s41598-018-28694-2 (PMC6039510; doi:10.1038/s41598-018-28694-2)
Supplement: Supplementary file 3 — Supplementary Information and Protocol [file 41598_2018_28694_MOESM3_ESM.pdf]

Supplementary Information for

Volumetric, Nanoscale Optical Imaging of Mouse and Human Kidney via Expansion Microscopy

Tyler J. Chozinski[1], Chenyi Mao[1], Aaron R. Halpern[1], Jeffrey W. Pippin[2], Stuart J. Shankland[2], Charles E. Alpers[3], Behzad Najafian[3], Joshua C. Vaughan[1,4,*]

[1]Department of Chemistry, [2]Department of Medicine, Division of Nephrology, [3]Department of Pathology, and [4]Department of Physiology and Biophysics, University of Washington, Seattle, Washington, USA, 98195.

Correspondence: *J.C. Vaughan, Department of Chemistry, University of Washington, Box 351700, Seattle Washington, 98195, USA. E-mail: jcv2@uw.edu.*

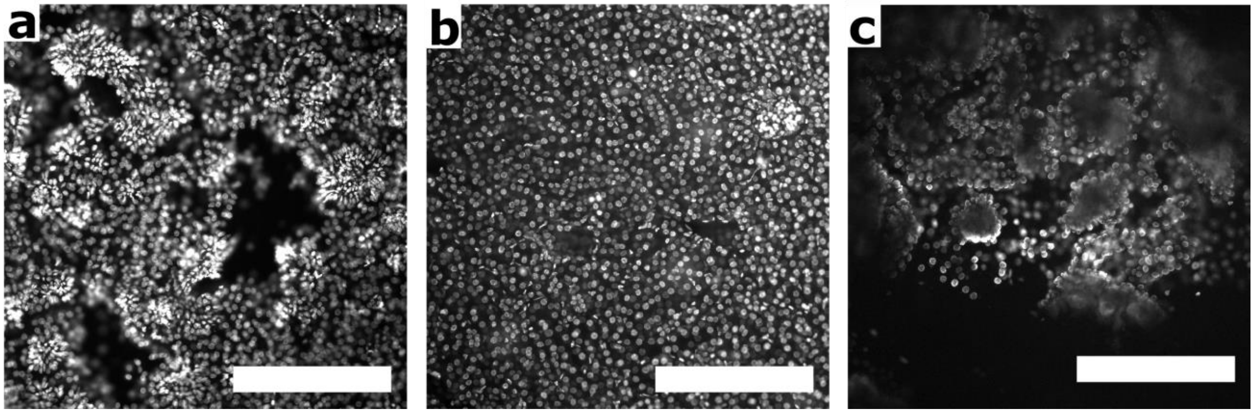

Supplementary Figure 1: Epifluorescence images of expanded 100 μm thick sections of mouse kidney stained for DNA with Hoechst and processed using different digestion protocols. (**a**) Mouse kidney tissue treated with MA-NHS for linkage of proteins to the hydrogel and digested with proteinase K alone leads to abundant tears (empty areas) and under-expanded regions exhibiting relatively small or stretched nuclei. (**b**) MA-NHS-treated tissue that was digested with proteinase K followed by collagenase leads to uniform sample homogenization and lacks obvious distortions. (**c**) MA-NHS-treated tissue that is later homogenized with detergent and heat (MAP homogenization) leads to obvious tearing and distortions of the expanded specimen. All scale bars are in pre-expansion units. Scale bars: 200 μm.

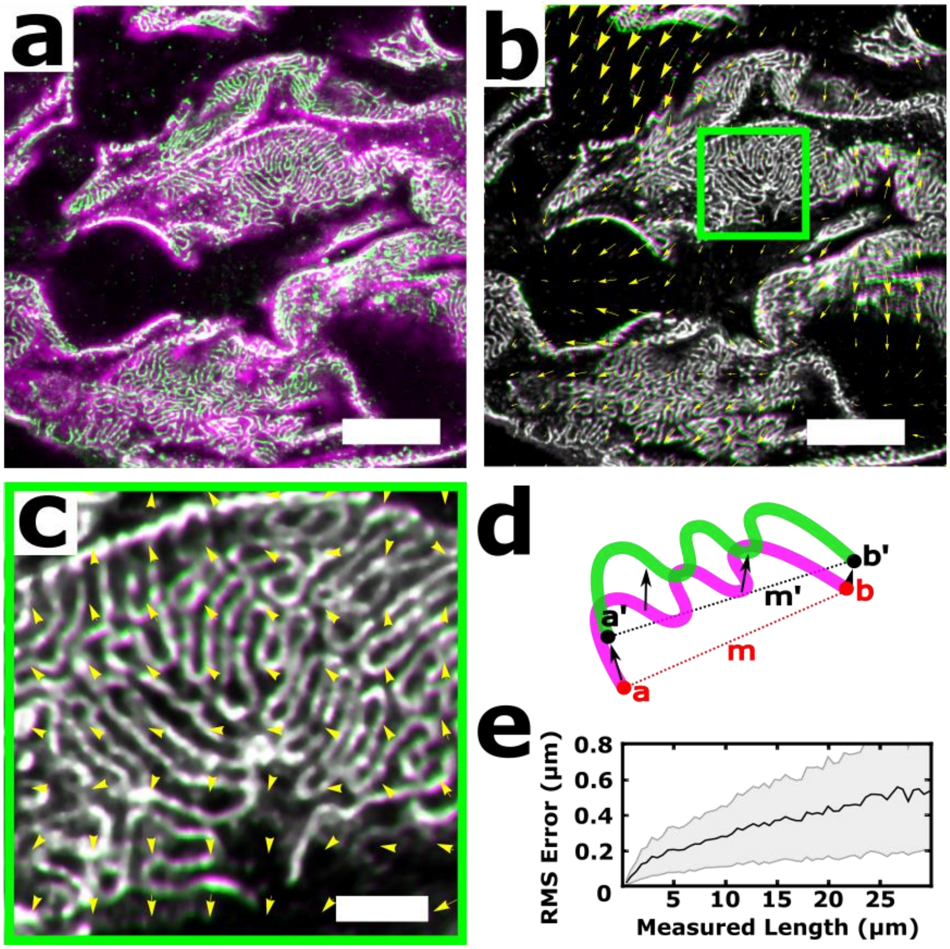

Supplementary Figure 2: Comparison of pre- and post-expansion confocal images of expanded mouse kidney tissue stained for podocin (data from **Fig. 1**). (**a**) Overlay of pre-expansion (magenta) and post-expansion (green) images after registration by similarity transform (a rigid transformation including scaling, rotation, and translation). A Gaussian blurring filter was applied to the post-expansion image prior to registration in order for the pre- and post-expansion images to have similar "resolution". (**b**) Overlay of post-expansion image before (magenta) and after (green) the application of a B-Spline registration (non-rigid transformation) to warp the post-expansion image so that it aligns to the pre-expansion image (considered to be ground truth data). Yellow arrows show the direction and relative magnitude (scaled up by a factor of 8 for visibility) of the local transformation required to align the post-expansion image to the pre-expansion image. (**c**) Zoomed view of the boxed region in **b**. Note that in both **b** and **c** the white color indicates that the magenta and green channels overlap. (**d**) Schematic of correlation analysis procedure. For a detailed distortion analysis protocol, see Chozinski *et al.* 2016.[1] Briefly, post-expansion images before and after B-spline registration are binarized to create "skeletons" (green and magenta lines in **d**). 50,000 points are then randomly chosen from each skeleton and the distance between all points in each image is calculated. The value *m* represents the distance between points *a* and *b* in the pre-B-Spline-registration image (magenta) while *m′* is the distance between *a′* and *b′* in the post-B-Spline-registration image (green). RMS error plots (**e**) were generated by calculating the difference between *m* and *m′* as a function of distance *m* for 25,000 sets of points throughout the image (shaded areas represent plus or minus the standard deviation). Note that **e** was calculated by performing the distortion analysis in three dimensions. All distances and scale bars are in pre-expansion units. Scale bars: 5 µm (**a, b**) and 1 µm (**c**).

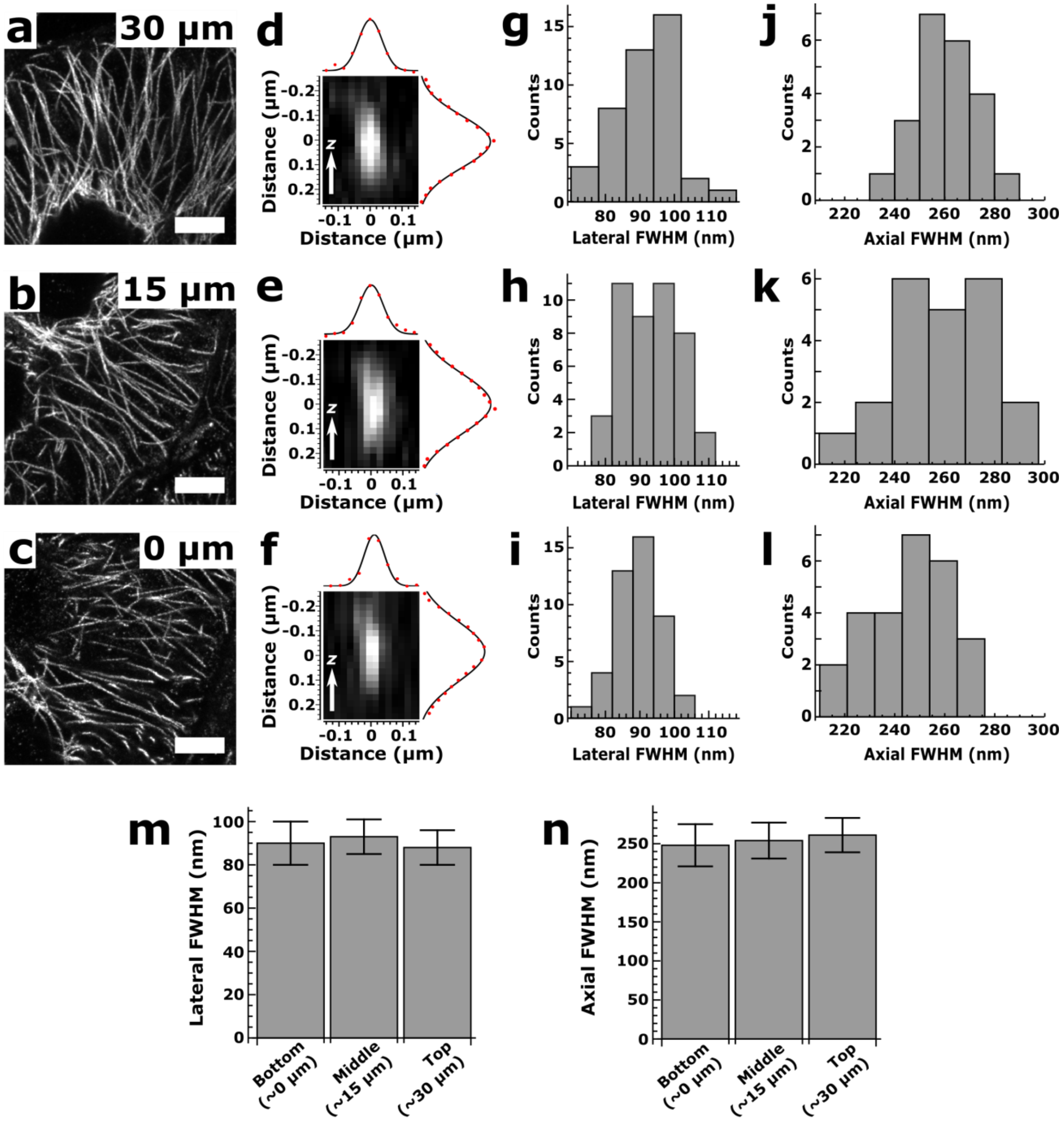

Supplementary Figure 3: Estimation of resolution using cross-sectional profiles of microtubules in expanded mouse kidney. (**a-c**) Confocal fluorescence images (representative ~500 nm thick projections taken from 5 µm stacks) of expanded mouse kidney immunostained for tyrosinated tubulin taken at 0, 15, and 30 µm depths (which corresponds to 0 µm, 60 µm, and 120 µm in post-expansion dimensions and is near the limit of the objective lens's working distance). (**d-f**) Representative cross-sections of individual microtubules at the same depths corresponding to **a**, **b**, and **c** including cross-sectional profiles with Gaussian fits (lateral, top; axial, right). (**g-l**) Histograms of full width at half-maximum (FWHM) values of cross-sectional profiles of single microtubules fitted with Gaussian functions in lateral (**g-i**) and axial (**j-l**) dimensions. (**m**) Summary of histograms in **g**, **h**, and **i** showing average lateral FWHM values of **g** 88 ± 8 nm (mean ± SD, 47 profiles), **h** 93 ± 8 nm (mean ± SD, 44 profiles), and **i** 90 ± 10 nm (mean ± SD, 45 profiles).

Using our previous study[1] where the convolution of a double-peaked cross-sectional profile of indirectly immunostained microtubules measured by localization microscopy (at ~20 nm resolution) with a ~65 nm Gaussian point spread function (the estimated resolution) yielded an observed microtubule FWHM of ~80 nm, we estimate our lateral resolution here to be 70-75 nm. (**n**) Summary of histograms in **j**, **k**, and **l** showing average axial FWHM values of **j** 261 ± 13 nm (mean ± SD, 47 profiles), **h** 254 ± 23 nm (mean ± SD, 23 profiles), and **i** 248 ± 19 nm (mean ± SD, 27 profiles). From this data, we estimate our axial resolution to be ~250 nm. The expansion protocol effectively clears the tissue and the hydrogel changes the sample's refractive index to that of water. By using a water immersion objective lens, spherical aberration due to refractive index mismatch is minimized and, as shown here, the resolution is preserved throughout the thickness of the sample. All distances and scale bars correspond to pre-expansion dimensions. Scale bars: 3 µm.

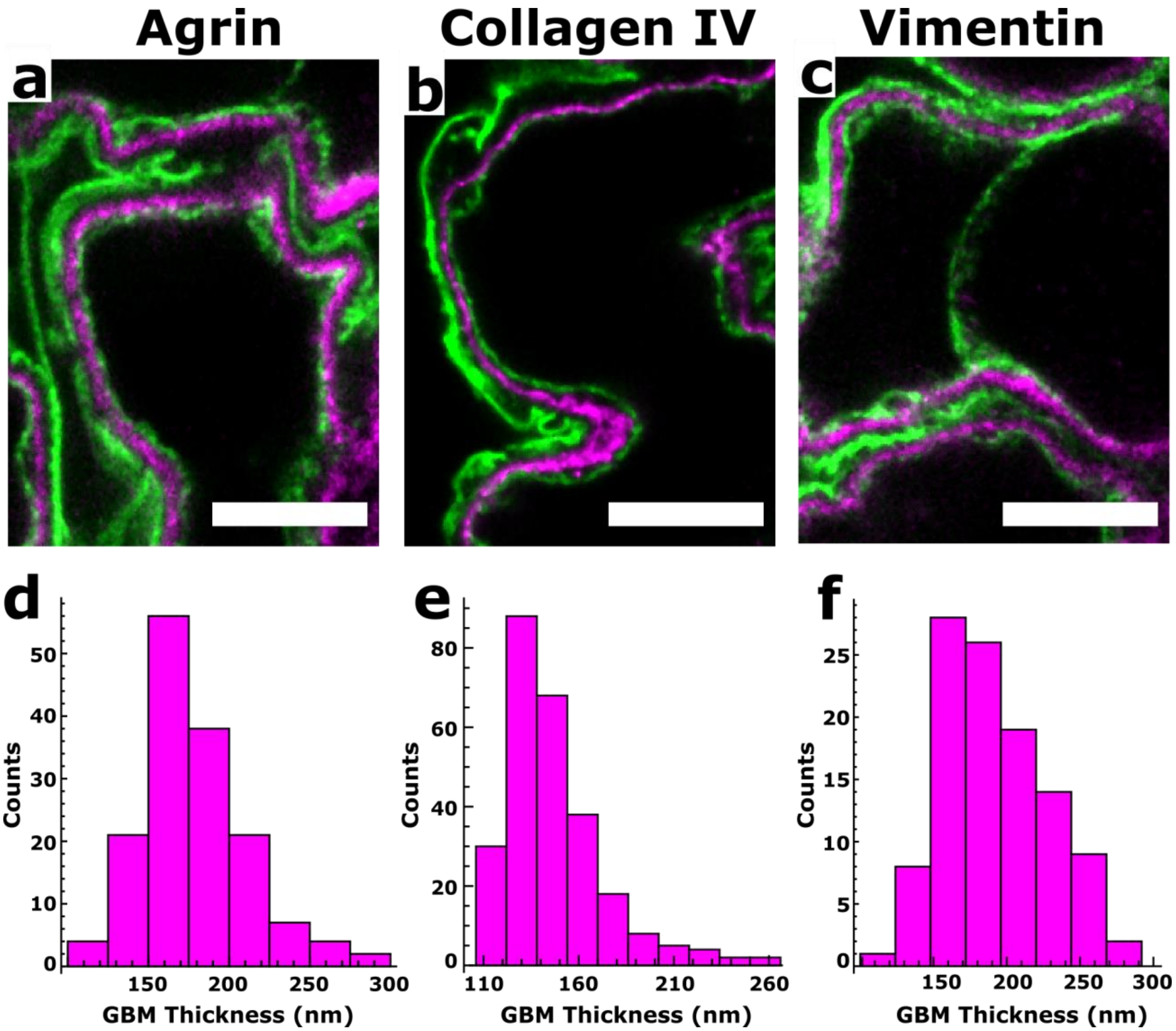

Supplementary Figure 4: Glomerular basement membrane (GBM) markers and GBM thickness measurements from confocal microscopy (orthogonal sections) of expanded 100 µm thick slices of mouse kidney. (**a-c**) Immunostaining of podocalyxin (green) and various GBM markers (magenta): agrin (**a**), collagen IV (**b**), and vimentin (**c**). (**d**-**f**) Distributions of GBM thicknesses measured in agrin (**a**), collagen IV (**b**), and vimentin (**c**) with average values of 178 ± 34 nm, 147 ± 26 nm, and 192 ± 36 nm, respectively (mean ± SD). The number of samples used in each case is as follows: agrin (3 kidney samples, 4 glomeruli, 154 cross-sectional profiles), collagen IV (3 kidney samples, 7 glomeruli, 263 cross-sectional profiles), and vimentin (3 kidney samples, 3 glomeruli, 107 cross-sectional profiles). All distances and scale bars are in pre-expansion units. Scale bars: 3 µm.

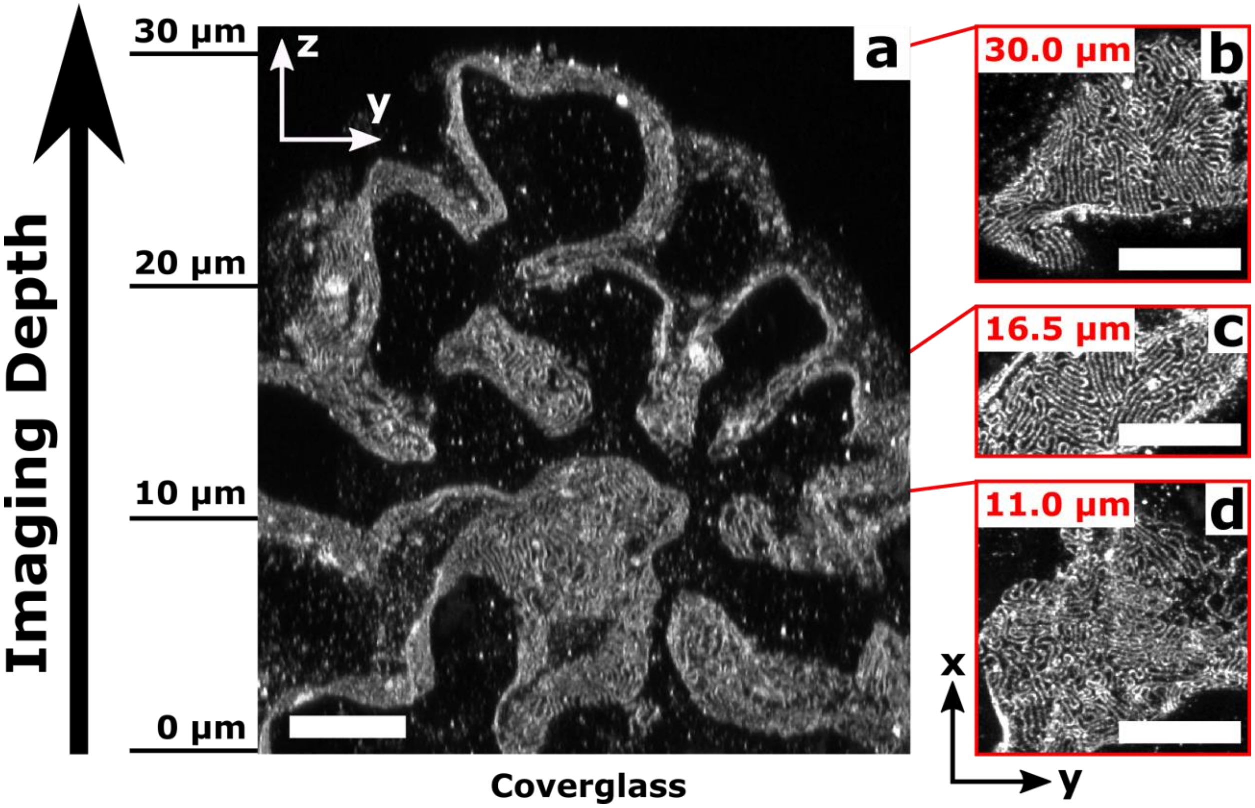

Supplementary Figure 5: Confocal images of expanded mouse kidney stained for podocin. (**a**) Confocal maximum intensity projection of 2.5 µm thick section in the YZ plane showing an imaging depth of ~30 µm (which corresponds to ~120 µm in post-expansion dimensions and is near the limit of the objective lens's working distance). Note that foot process boundaries are still clearly resolvable in the *z*-dimension throughout the entire imaging depth. (**b**-**d**) Confocal maximum intensity projections of 1.4 µm (**b**), 1.3 µm (**c**), and 1.8 µm (**d**) thick sections in the XY plane at various imaging depths. All distances and scale bars are in pre-expansion units. Scale bars: 5 µm. See also **Supplementary Movie 2** for an animation of this volume.

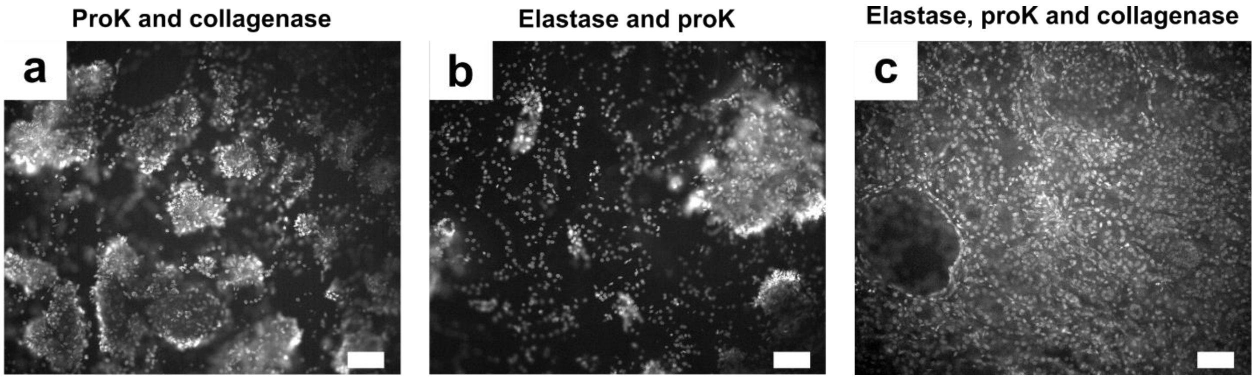

Supplementary Figure 6: Epifluorescence images of expanded 100 µm thick sections of human kidney stained for DNA with Hoechst and processed for expansion using different enzymatic treatments to homogenize the tissue. Enzymatic digestion with proteinase K and collagenase (**a**) or elastase and proteinase K (**b**) leads to an incomplete digestion with obvious tears and tissue loss, whereas digestion with elastase, proteinase K, and collagenase (**c**) enables uniform expansion. Scale bars are all 100 µm and are in pre-expansion dimensions.

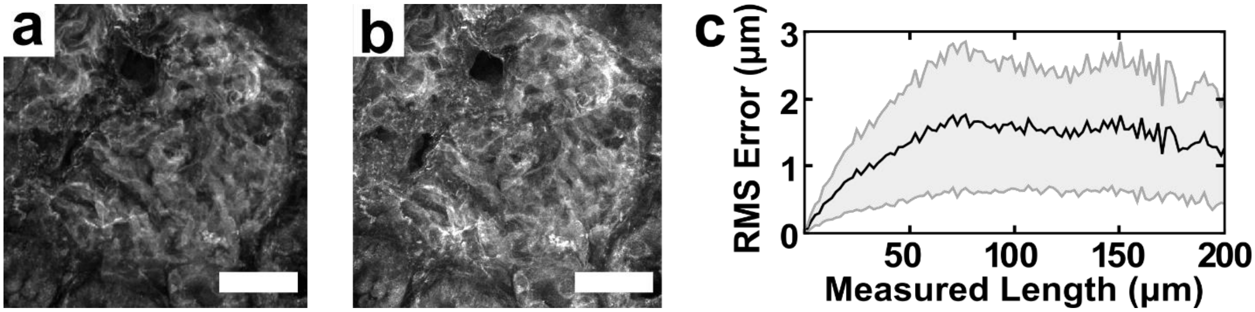

Supplementary Figure 7: Comparison of pre-expansion **(a)** and post-expansion **(b)** images of human kidney tissue taken by confocal fluorescence microscopy for a whole glomerulus immunostained for podocin. (**c**) Quantification of root mean square (RMS) error of pre- versus post- expansion confocal images of the human kidney tissue. All distances and scale bars are in pre-expansion units. Scale bars are 50 μm.

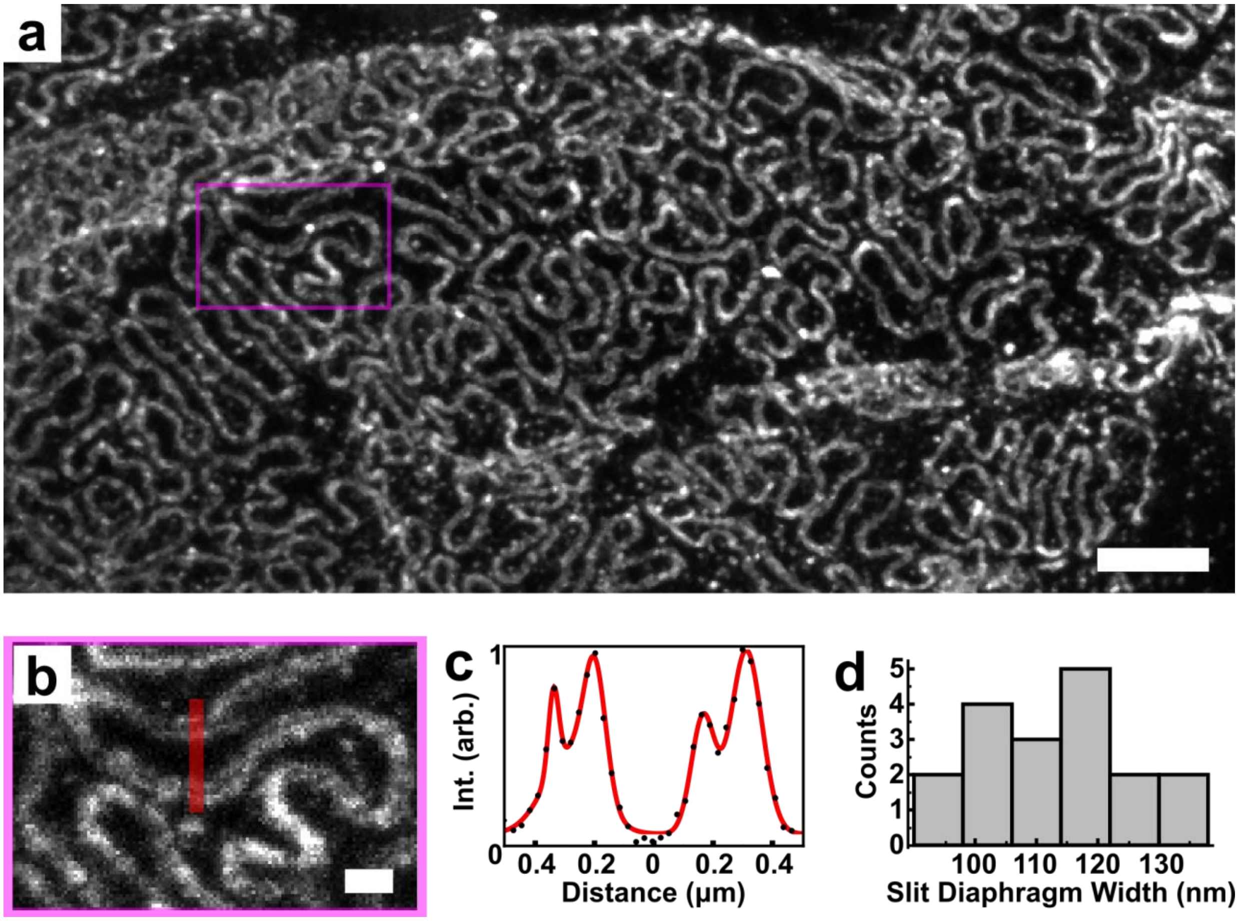

Supplementary Figure 8: Confocal fluorescence images of expanded human kidney tissue. (**a**) Human kidney immunostained for podocin using the conventional antibody. (**b**) zoomed-in view of the boxed area in **a**, showing resolvable gap between adjacent podocytes. (**c**) Cross-sectional profile of red line in **b** fitted with multiple Gaussian functions. (**d**) Histogram of measured width between adjacent podocin signals (taken to be the slit diaphragm) in **a**. Scale bars are 2 μm (**a**) and 500 nm (**b**) and are all in pre-expansion dimensions.

Supplementary Movie 1. Animated rendering of data from **Fig. 3a** showing a 28×28×11 μm^3 volume (tick marks spaced by 420 nm) of mouse kidney tissue immunostained for podocin (green), agrin (red), podocalyxin (blue), and stained for DNA with Hoechst (white). All distances and units are in pre-expansion units.

Supplementary Movie 2. Animated rendering of data from **Supplementary Fig. 4** showing a 28×28×31 μm^3 volume (tick marks spaced by 550 nm) of mouse kidney tissue immunostained for podocin. All distances and units are in pre-expansion units.

Supplementary Table 1. Summary of sample preparation and imaging conditions. All primary antibody incubations were 18-24 hours at 25 °C at a concentration of 1-2 µg/mL and all secondary antibodies were incubated for 18-24 hours at 25 °C at a concentration of 2.5-5 µg/mL. The dye-to-IgG ratios for secondary antibodies were as follows: Alexa 488, 8-10 dyes/IgG; Alexa 568, 5-7 dyes/IgG; Atto 647N, 2-3 dyes/IgG.

**Fig.**	**Specimen Info**	**1° Ab**	**2° Ab, etc.**	**Digestion†**	**Imaging Info**<br>*Distances in pre-expansion units
1b-c, e-f,h-i, S2	C57/BL6 mouse, drop-fixed in 4% PFA for 1 hr, 100 µm vibratome section	Rb x Podocin	D x Rb Alexa 488	ProK overnight, coll overnight	Confocal: **b-c**) 10x 0.4NA, 40µm thick max projections; **e,h,S2**) 63x 1.2NA,~500nm single plane; **f**,**i**,**S2**) 63x 1.2NA, ~130nm single plane
2a-d, 3a-c,g,I, Supp. Movie 1	C57/BL6 mouse, drop-fixed in 4% PFA for 1 hr, 100 µm vibratome section	Rb x Podocin<br>Ms x Agrin<br>G x Podocalyxin	D x Rb Alexa 488<br>D x Ms Alexa 568<br>D x G Atto 647N	ProK overnight, coll overnight	Confocal 63x 1.2 NA; **2a-d**) ~130 nm single confocal plane; **3a**) 11 µm thick (in *z*) 3D rendering; **3b-c**) ~700 nm max projection; **3g,i**) ~130 nm single plane
2e-h	C57/BL6 mouse, drop-fixed in 4% PFA for 1 hr, 100 µm vibratome section	GP x Synaptopodin<br>Ms x acet. Tubulin<br>Rb x Podocin	D x GP Atto 647N<br>D x Ms Alexa 568<br>D x Rb Alexa 488	ProK overnight, coll overnight	Confocal 63x 1.2 NA, 1.4 µm max projections
2i-l	C57/BL6 mouse, drop-fixed in 4% PFA for 1 hr, 100 µm vibratome section	Rb x Collagen IV<br>G x Podocalyxin<br>Ms x α SMA	D x Rb Alexa 488<br>D x G Atto 647N<br>D x Ms Alexa 568	ProK overnight, coll overnight	Confocal 20x 0.7 NA, ~30 µm max projections
2m-r	Podocyte-confetti mouse, fresh frozen in OCT, 40 µm cryosection drop fixed in 4% PFA for 1 h	No stains; only fluorescent proteins were used (GFP, YFP, and RFP)	None	ProK overnight, coll overnight	Confocal 63x 1.2 NA: ~130 nm single plane
4, S7, S8	Human nephrectomy tissue, drop-fixed in 4% PFA for 1 hr, 100 µm vibratome section	Rb x Podocin	D x Rb Atto 488<br>Hoechst	Elast overnight, proK two days at 65°C, and coll overnight	Confocal: **4**) 63x 1.2 NA, ~1.15 µm max projection; **S6**) 20x 0.7 NA, ~9.1 µm max projection; **S7**)
S6	Human nephrectomy tissue, drop-fixed in 4% PFA for 1 hr, 100 µm vibratome section	None	Hoechst (nuclei)	**a**) ProK 2 days at 65 °C, coll overnight<br>**b**) Elast overnight, proK 2 days at 65 °C<br>**c**) Elast overnight, proK 2 days at 65 °C, coll overnight	Widefield epifluorescence image, 4× 0.2 NA air objective
S1a-c	C57/BL6 mouse, drop-fixed in 4% PFA for 1 hr, 100 µm vibratome section	None	Hoechst (nuclei)	**a**) ProK overnight<br>**b**) ProK overnight, coll overnight<br>**c**) 200 mM SDS, 200mM NaCl, 50mM tris pH 9, 24 h at 70 °C then 24 h at 90 °C	Widefield epifluorescence image, 4× 0.2 NA air objective
S3	C57/BL6 mouse, drop-fixed in 4% PFA for 1 hr, 100 µm vibratome section	Rat x Tubulin	D x Rat Atto 488	ProK overnight, coll overnight	Confocal: 63x 1.2 NA, ~500 nm max projections
S4	C57/BL6 mouse, drop-fixed in 4% PFA for 1 hr, 100 µm vibratome section	**a-c**) G x Podocalyxin<br>**a**) Ms x Agrin<br>**b**) Rb x Collagen IV<br>**c**) Ms x Vimentin	**a-c**) D x G Atto647N<br>**a**) D x Ms Alexa 568<br>**b**) D x Rb Atto 488<br>**c**) D x Ms Alexa 568	ProK overnight, coll overnight	Confocal: 63x 1.2 NA, ~130 nm single confocal plane
S5, Supp. Movie 2	C57/BL6 mouse, drop-fixed in 4% PFA for 1 h, 100 µm vibratome section	Rb x Podocin	D x Rb Atto 488	ProK overnight,coll overnight	Confocal, 63x 1.2 NA, max projections with thicknesses in µm of: **a**) 2.5; **b**) 1.4; **c**) 1.3; **d**) 1.8.

† Enzymatic digestion steps performed at 37 °C except as indicated otherwise; proK, coll, and elast are short for proteinase K, collagenase, and elastase, respectively

References:

1. Chozinski, T. J. *et al.* Expansion microscopy with conventional antibodies and fluorescent proteins. *Nat. Methods* **13,** 485–488 (2016).

Supplementary Protocol 1 – Expansion of Mouse and Human Kidney Tissue Sections

BACKGROUND: This protocol is based on methods described in the publication "Expansion microscopy with conventional antibodies and fluorescent proteins" (DOI: 10.1030/nmeth.3833)[1]; however, it has been adapted for expansion of mouse and human kidney tissue that has been fixed for 1 h in 4% paraformaldehyde. Other tissues or fixation conditions may require a different procedure. In general it is best to start with bright, robust stains to assess effectiveness of expansion, and to use relatively small specimens (e.g., ~1-2 mm^2 × 100 µm thick). It is also important to assess possible distortions by imaging the same specimen before and after expansion (with both high and low magnification). A detailed procedure for analyzing distortions in pairs of pre-expansion/post-expansion images is described in the publication listed above.

REAGENTS:

1. Ammonium Persulfate (APS is a salt and initiates polymerization)
    a. Store at 4 °C
2. Tetramethylethylenediamine (TEMED is a liquid and catalyzes polymerization)
    a. Store at 4 °C
3. 2,2,6,6-tetramethylpiperidine 1-oxyl (TEMPO is a solid and inhibits polymerization so that gelation starts after reagents permeate sample)
    a. Store at 4 ^{0}C
4. Methacrylic Acid-NHS (MA-NHS is a solid and is used to link proteins to the gel)
    a. Store the powder at 4 °C, <u>KEEP AWAY FROM WATER</u>
    b. Allow to warm to room temperature before opening to avoid condensation.
5. 40% Acrylamide (w/v) (Acrylamide is a liquid solution and is a monomer of the hydrogel)
    a. Store at 4 °C
6. 2% Bisacrylamide (w/v) (Bisacrylamide is a liquid solution and is a hydrogel crosslinker)
    a. Store at 4 °C
7. Sodium Acrylate (SA is an ionic monomer for the hydrogel)
    a. Store at room temperature, dry
8. Sodium Chloride (Salt)
9. 10x PBS Buffer
10. 10x TAE Buffer (Tris base, acetic acid, and EDTA)
11. 8 M Guanidine-HCl (component of digestion buffer)
12. Proteinase K (digestion enzyme, 600-800 Units/mL in glycerol. We typically purchase stocks from NEB.)
    a. Store at -20 °C
13. Collagenase (digestion enzyme, ≤10 units/mg solid. Sigma-Aldrich Blend Type F.)
    a. Store at -20 °C

STOCKS

1. APS: 10% (w/w) in water
    a. Store at -20 °C for up to 1 week
2. TEMED: 10% (v/v) in water
    a. Store at -20 °C for up to 1 week
3. TEMPO: 1% (w/w) in water
    a. Prepare freshly, within a few hours of use
4. MA-NHS: 1 M in anhydrous DMSO
    a. Store at -20 °C
    b. Keep away from water
5. Monomer Solution. Final concentrations are listed. Recipe achieves ~4x expansion. Bolded quantities in brackets, below, are for 10 mL of monomer solution.
    a. 1x PBS **[1 mL]**
    b. 2 M NaCl **[1.17 g]**
    c. 8.625% (w/v) Sodium Acrylate **[0.863 g]**
    d. 2.5% (w/v) Acrylamide **[0.625 mL]**
    e. 0.15% (w/v) Bisacrylamide **[0.75 mL]**
    f. Store at 4 °C for up to 1 month
6. Proteinase K digestion buffer. Bolded quantities in brackets, below, are for 10 mL of solution.
    a. 1x TAE Buffer **[1 mL]**
    b. 0.8 M Guanidine-HCl **[1 mL]**
    c. 0.5% Triton **[0.25 mL]**
    d. Store at 4 °C. Solution should be stable but we usually consume within a week.

7. Collagenase digestion buffer. Bolded quantities in brackets, below, are for 10mL of solution.
    a. 1M $CaCl_2$ **[7 µL]**
    b. 1X HBSS **[10 mL]**
    c. Store at 4 °C. Solution should be stable but we usually consume within a week.

POST-STAIN TREATMENT

1. After immunostaining the sample (or after expression of FP, **Figure 1A**), treat with 1 mM MA-NHS in PBS (diluted from your DMSO stock). Because NHS compounds rapidly hydrolyze in water, do not make the NHS solution in PBS until you are ready to treat your sample.
    a. Depending on how your tissue was fixed, you may need to alter the concentration of MA-NHS.
2. Allow the sample to react for 1 h at room temperature.
3. Wash the sample 2-3 times with several volumes of PBS.

GELATION

1. Incubate the tissue in monomer solution for 30-45 min at 4 °C prior to gelation to allow monomer to penetrate the whole tissue. **NOTE: the monomer here DOES NOT contain APS, TEMED, or TEMPO.**
2. Place tissue on #1.5 coverglass and remove excess monomer (wick away with Kim wipe). Ensure tissue is flat against the glass.

3. Prepare the gelation solution. Quantities in brackets, below, are for a 100 µL volume. Note that APS should always be added last, right before adding to the specimen.
    a. 0.2% TEMED **[2 µL of 10% solution]**
    b. 0.01% TEMPO **[1 µL of 1% solution]**
    c. 95% monomer solution **[95 µL of monomer stock]**
    d. 0.2% APS **[2 µL of 10% solution]**
4. Cover the tissue with the gelation solution without disturbing it. Avoid letting the tissue fold over or float up into the gel (**Figure 1B**).
5. Place two pieces of #1.5 coverglass stacked on either side of the tissue and cover with another #1.5 coverglass. Place a drop of leftover gelation solution on top of the coverglass as a "tester" gel to see when the polymerization is complete.
    a. We use 2 pieces of coverglass to make the resulting gel thicker so it's easier to handle. You can alter the number of pieces or use thinner glass for thinner gels. You'll figure out what works best when you try to image the expanded sample and find out your needs based on the working distance of your objective lens.
    b. If you're using a thick piece of tissue you may need to use thicker coverglass spacers to ensure the whole sample is incorporated into the gel.
6. Allow the sample to gel at 37 °C for 1.5-2 h.

DIGESTION

1. As described, below, the digestion of mouse kidney tissue sections consists of digestion with proteinase K and collagenase, while the digestion of human kidney tissue sections consists of digestion with elastase, proteinase K, and collagenase.
2. Remove top piece of coverglass as well as spacers. Cut away excess gel from around the tissue (Your sample will be cleared after expansion and will be difficult to find in a lot of excess gel.) Use a diamond knife or razor blade to score the bottom coverglass near to the tissue and then break away excess coverglass so that the coverglass and gel will fit into the digestion buffer well. Do not try to remove the gels from the coverglass because they may tear (**Figure 1C**).
3. Place the gel sitting on the coverglass in a suitably sized well, e.g., a well of a 12-well plate (**Figure 2A**).
    a. Keep in mind that the gels will expand slightly (~1.5x) during digestion so make sure the well is a bit larger than the gel.
4. **For mouse kidney section digestion**, use the following protocol for digestion.
    a. Add proteinase K digestion buffer with ~8 Units/mL of proteinase K to the sample. Make sure to cover the sample completely and allow to digest at 37 °C for 12-18 hours (typically done overnight).
    b. Wash the digested specimen three times with several volumes of PBS. Then add collagenase digestion buffer containing 5 mg/mL collagenase and incubate at 37 °C for 12-18 hours (typically done overnight).
5. **For human kidney section digestion**, use the following protocol for digestion.
    a. Digest in 1mg/mL elastase in 200mM Tris buffer (pH 8) at 37 ˚C for 12-18 hours (typically done overnight).
    b. Digest in ~8 Units/mL of proteinase K for two days at ~65 ˚C. Replace the digestion solution (digestion buffer and proteinase K) after the first day.

c. Wash the digested specimen three times with several volumes of PBS. Then add collagenase digestion buffer containing 5 mg/mL collagenase and incubate at 37 °C for 12-18 hours (typically done overnight).

EXPANSION

1. Remove gels from collagenase digestion buffer and place in DI water to expand. Anticipate the size of the expanded gel and use a suitably sized container (we often use a 3.5” petri dish).
2. Exchange water as needed until fully expanded (typically 2-3 exchanges). The refractive index of the gel is nearly identical to that of water so you will not easily see the gel. Be careful not to pour out or aspirate the gel. Typical expansion times are 1-2 hours, total, with exchanges every 30 min (**Figure 2B-C**).
3. Thinner gels will expand relatively quickly and may only need one water exchange.

SAMPLE HANDLING TIPS

- Removing expanded gels from petri dishes (or handling them in general) can be difficult. A large rectangular coverglass (~1” x 2”) is probably the best tool to use but other flat objects or spatulas may also work well. Place the coverglass short edge against the petri dish surface and tilt the dish to allow the gel to gently slide onto the coverglass.
- Try to gently wick away excess water before imaging using a Kim wipe. The gels will otherwise slide around during imaging.
- If the gels fold over onto themselves after removing excess water, try to use a fine-tipped paintbrush and gently poke the edges of the gel back until the sample is back in its original shape.
- A flashlight (illuminating from below) and a dark background are helpful when trying to locate your tissue within the gel. Look for a small amount of scattering.
- To prevent gels from sliding during imaging, mount them on poly-lysine-coated coverglass. Do this by sliding the gel from a spatula (or whatever tool was used to pick up the gel) onto the coated glass until the first edge sticks down. Continue sliding the spatula out from under the gel allowing it to slowly sit down on the coated glass so that the gel does not bend.

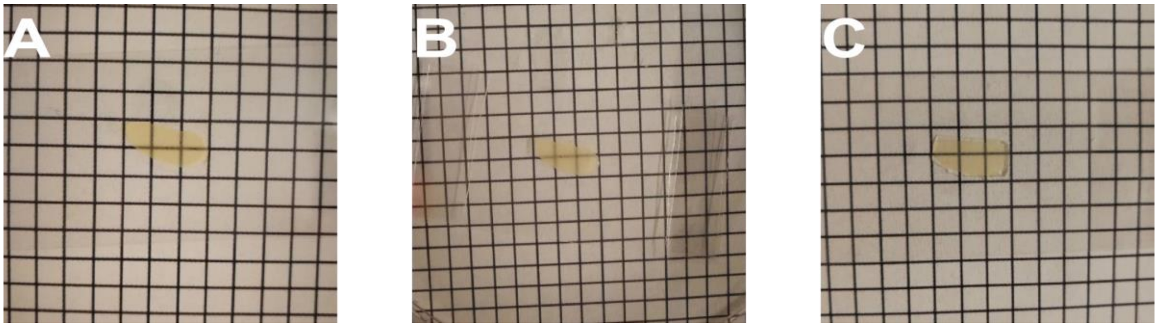

Figure 1. A) A flat, 100 µm mouse kidney section, after immunostaining ,was mounted on a coverglass. **B**) The MA-NHS treated section was polymerized in a home-built chamber consisting of two coverglasses

with a spacer as described in the text. **C**) After polymerization, the excess gel around the specimen was trimmed with a razor blade. The coverglass substrate was also trimmed with a glass cutter or razor blade to enable it to fit in a 12-well plate for digestion. The grid lines are separated by ~4mm.

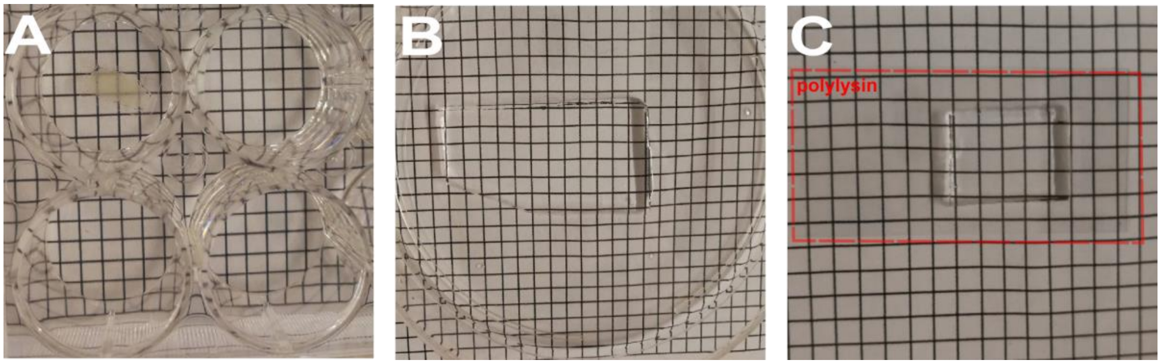

Figure 2. A) Trimmed hydrogel specimen was placed in a well of a 12-well plate to digest. **B**) Specimen after expansion. **C**). Portion of expanded hydrogel mounted on a polylysine-coated coverglass. The grid lines are separated by ~4 mm.

Reference

(1) Chozinski, T. J.; Halpern, A. R.; Okawa, H.; Kim, H.-J.; Tremel, G. J.; Wong, R. O. L.; Vaughan, J. C. Expansion Microscopy with Conventional Antibodies and Fluorescent Proteins. *Nat. Methods* **2016**, 12, 483-488.
